# Evaluation of antioxidant and anticancer activity of extract and fractions of *Nardostachys jatamansi* DC in breast carcinoma

**DOI:** 10.1186/s12906-015-0563-1

**Published:** 2015-03-10

**Authors:** Shilpee Chaudhary, Kodangala Subraya Chandrashekar, Karkala Sreedhara Ranganath Pai, Manganahalli Manjunath Setty, Raviraj Anand Devkar, Neetinkumar Dnyanoba Reddy, Muhammed Haneefa Shoja

**Affiliations:** Department of Pharmacognosy, Manipal College of Pharmaceutical Sciences, Manipal University, Manipal, Karnataka 576 104 India; Department of Pharmacology, Manipal College of Pharmaceutical Sciences, Manipal University, Manipal, Karnataka 576 104 India

**Keywords:** *Nardostachys jatamansi*, Breast cancer, MCF-7, MDA-MB-231, Antioxidant, Cell cycle, Apoptosis, Clonogenic assay, Lupeol, Beta-sitosterol

## Abstract

**Background:**

*Nardostachys jatamansi* DC is a Himalayan medicinal herb that has been described in various traditional systems of medicine for its use in cancer. In view of its traditional claims, and chemical constituents, antioxidant and anticancer activities were evaluated in breast carcinoma.

**Methods:**

Petroleum ether (NJPE), methanol extract (NJM) and subsequent diethyl ether (NJDE), ethyl acetate (NJEA) and aqueous (NJAQ) fractions of roots and rhizomes of *N. jatamansi* were prepared. Total phenolic, flavonoid content, and antioxidant activities were determined using suitable methods. Antiproliferative activity was assessed in estrogen receptor (ER)-positive (MCF-7) and ER-negative breast carcinoma (MDA-MB-231) cells by MTT and SRB assay. Cell cycle analysis, Hoechst staining, and clonogenic assay were employed to determine the mode of antiproliferative and pro-apoptotic activity in MDA-MB-231 cells.

**Results:**

NJM/fractions exhibited prominent antioxidant activity with significant correlation between phenolic content and ABTS (IC_50_) scavenging (R = −0.9680, *P* < 0.05), and total antioxidant capacity (R = 0.8396, *P* > 0.05). In MTT assay, NJM exhibited the highest antiproliferative activity (IC_50_: 58.01 ± 6.13 and 23.83 ± 0.69 μg/mL in MCF-7 and MDA-MB-231 respectively). Among the fractions, NJPE and NJDE were found to be most potent in MCF-7 (IC_50_: 60.59 ± 4.78 μg/mL) and MDA-MB-231 (IC_50_: 25.04 ± 0.90 μg/mL) cells respectively. Statistical analyses revealed NJM and NJDE exhibited significantly higher (*P* < 0.05) cytotoxicity in MDA-MB-231 cells. Cell cycle analysis demonstrated that NJM, NJPE and NJEA caused G_2_/M arrest while NJDE caused G_0_/G_1_ phase arrest in MDA-MB-231 cells. Further, NJM/fractions induced significant (*P* < 0.001) cell death by apoptosis characterized by apoptotic morphological changes in Hoechst staining and inhibited long-term proliferation (*P* < 0.001) of MDA-MB-231 cells in clonogenic assay. Lupeol and β-sitosterol were identified as anticancer principles in NJM/fractions by HPTLC.

**Conclusion:**

Our results suggest that NJM/fractions possess significant antiproliferative potential which is mediated through cell cycle perturbation and pro-apoptotic effects in MDA-MB-231 cells. Moreover, this study highlights the antioxidant potential of NJM/fractions which can be attributed to the presence of phenols. NJDE emerged as the most potent fraction and further mechanistic and phytochemical investigations are under way to identify the active principles.

## Background

Breast cancer has traditionally been less common in developing countries; however, industrialization is associated with rapid increase in breast cancer risk. Environmental factors including diet and exposure to free radicals play a critical role in the development of breast cancer. With the advent of selective estrogen replacement modulators (SERMs) and aromatase inhibitors, there has been a significant impact on morbidity and mortality in estrogen receptor (ER) positive breast cancer however; these drugs pose a number of serious adverse effects like uterine cancer, thromboembolism, cataracts, and perimenopausal symptoms [1-3]. On the other hand, the more malignant and aggressive ER negative breast cancer is unresponsive to hormonal therapy and current therapeutic modalities are associated with toxicity and side effects [4].

Given the current state of research and poor prognosis of breast cancer, novel therapeutics is needed to be developed as potential anti-tumor agents. Aberrant cell cycle progression and resistance to apoptotic cell death are hallmarks of cancer cells. Cancer cell populations lose their ability to negatively regulate cell cycle leading to uncontrolled cellular proliferation and acquire the potential to evade apoptosis consequently, diminished cell attrition [5]. These are two of the most important factors that are conducive to cancer progression. A plethora of evidence from previous studies suggest that a wide range of chemicals derived from medicinal plants exhibit significant anticancer activity by modulating signaling cascades associated with cell cycle regulation and/or apoptosis [6,7]. Since apoptosis and cell cycle arrest are essentially involved in neoplastic transformation and metastases, activation of apoptotic pathways and negative regulation of cell cycle progression in cancer cells are considered to be valuable therapeutic approaches for cancer treatment.

In addition, early modulation of oxidative stress by exogenous antioxidants like curcumin, resveratrol, catechin, gensitein, and consumption of diet rich in vitamins and antioxidants has proven beneficial in cancer prevention [8,9]. Plant polyphenols are an important class of compounds that act on multiple cancer-inflammation and reactive oxygen/reactive nitrogen species (ROS/RNS)-mediated pathways that inhibit oxidative stress and DNA damage that is implicated in mutagenesis, carcinogenesis, and premature ageing [8-11]. Free radicals react with purines, pyrimidines, and chromatin protein leading to base modifications, unstable genomes, and genetic alterations. These transformed cells have altered levels of cell cycle and apoptosis signaling molecules thereby resulting in uncontrolled cell proliferation and tumor formation [12]. Hence, preventing ROS/RNS-mediated damage to cellular biomolecules has emerged as an attractive strategy for preventing cancer [13,14].

*Nardostachys jatamansi* DC is a small, erect, hairy, endangered, perennial herb belonging to the family valerianaceae [15,16]. The roots and rhizomes are harvested throughout the Himalayas and traded from the alpine regions to the plains of India. The plant also grows in Nepal, Bhutan, South-West China, Afghanistan and Pakistan [17]. *N. jatamansi* has a long history of medicinal use which dates back to 1000–800 B.C. in Ayurveda and Unani systems of medicine [18]. The rhizomes are rich in sesquiterpenoids, terpenic coumarins, phenols, flavonoids, alkaloids, lignans, and neo-lignans [16,18-21]. The plant is described in the traditional systems of medicine for its use as sedative, antidepressant, antiepileptic, antihysteric, hypotensive, antispasmodic, anti-inflammatory, and cardiotonic [20]. The roots are considered aromatic, bitter tonic, antispasmodic, deobstruent, stimulant, antiseptic, diuretic, and emmenagogue [16]. The roots of the plant were also used traditionally for indurations and solid tumours in different systems of medicine [22,23]. Bhagat *et al*., have reported the cytotoxicity of alcoholic extract and n-butanol fraction of *N. jatamansi* in lung, liver, ovary and prostate cancer cell lines [24]. Moreover, two new sesquiterpenoids have been isolated from the roots and rhizomes of *N. jatamansi* and cytotoxicity of the crude chloroform:methanol extract and the isolates have been studied in lung, prostate, ER-positive breast cancer and neuroblastoma cell lines [23,25].

To our knowledge, this is the first study investigating the cytotoxic activity of the whole methanol extract and subsequent fractions of *N. jatamansi* in ER-positive (MCF-7) and ER-negative breast cancer (MDA-MB-231) cells simultaneously. We observed that *N. jatamansi* extract/fractions exhibited significantly higher cytotoxicity in MDA-MB-231 cells as compared to MCF-7 cells. Therefore, we explored the mode of action of antiproliferative activity of whole extract and fractions in MDA-MB-231 cells by studying the effect of *N. jatamansi* extract/fractions on cell cycle progression, apoptosis and clonogenic capacity of breast cancer cells.

In addition, the antioxidant potential of whole hydroalcoholic extract of *N. jatamansi* has been reported by DPPH, superoxide, hydroxyl radical scavenging and total antioxidant capacity assays [21] however, we report for the first time the antioxidant activity of extract and subsequent fractions of *N. jatamansi* by various antioxidant assays. A possible correlation was also investigated between the antioxidant activity and total phenolic and flavonoid content of the plant extract/fractions which would lay considerable evidence for its use as an adjuvant to mitigate oxidative stress in cancer progression.

## Methods

### Chemicals

Folin-Ciocalteu reagent, gallic acid, quercetin, ascorbic acid, curcumin, β-sitosterol, lupeol, 1,1-diphenyl-2-picrylhydrazyl (DPPH), 2,2′-azino-bis(3-ethylbenzothiazoline-6-sulfonic acid) diammonium salt (ABTS), 3-(4, 5-dimethylthiazolyl-2-yl)-2,5-diphenyl tetrazolium bromide (MTT), sulforhodamine B (SRB), Hoechst 33258 dye, crystal violet, propidium iodide were purchased from Sigma Chemicals Co. (St. Louis, MO, USA). All other chemicals and solvents were of analytical grade and purchased from the usual sources.

### Plant material

The roots and rhizomes of *N. jatamansi* were collected from a genuine crude drug supplier in Uttarakhand in the month of September, 2013. The plant was authenticated by Dr. K. Gopalkrishna Bhat, Professor and Head (Ret.), Department of Botany, Poornaprajna College, Udupi. A voucher specimen (PP 587) has been deposited in the herbarium of our institute, Department of Pharmacognosy, Manipal College of Pharmaceutical Sciences, Manipal for future reference.

### Preparation of extracts

Petroleum ether extract (NJPE) was prepared from the dried roots and rhizomes of *N. jatamansi*. Owing to the high percentage of essential oils in *N. jatamansi*, cold maceration was carried out with petroleum ether to rule out the possibility of any artifact formation at high temperature. The coarsely powdered drug (133 g) was extracted with petroleum ether by cold maceration for 5 days with occasional shaking. The extract was filtered and concentrated under reduced pressure and controlled temperature in a rotary evaporator and stored at 4°C in refrigerator for further use.

Conventional hot Soxhlet extraction was used to prepare methanol extract wherein, the coarsely powdered drug (318 g) was extracted with methanol at 68°C and evaporated under reduced pressure and controlled temperature to give a dark brown semisolid extract (NJM). The crude extract was suspended in water to afford an aqueous methanol solution and then partitioned with diethyl ether and ethyl acetate to yield diethyl ether (NJDE), ethyl acetate (NJEA) and remaining aqueous fraction (NJAQ).

### Phytochemical analysis

Preliminary phytochemical analysis of the extract/fractions was carried out for the presence of secondary metabolites *viz*. alkaloids, terpenoids, sterols, phenols, flavonoids, glycosides, tannins, saponins, fixed oils and fat by standard tests [26,27]

### Total phenolic content

Total phenolic content was estimated in extract and fractions of *N. jatamansi* using Folin-Ciocalteau reagent [28]. Gallic acid was used as standard. One mL of standard/extract solution was mixed with 5 mL Folin-Ciocalteu reagent (diluted ten times with water) and 4 mL of 0.7 M sodium carbonate. The absorbance was measured after incubation for 2 h at 765 nm with a UV-spectrophotometer. All determinations were carried out in triplicate. The concentration of phenolic compounds in the extracts was determined from gallic acid calibration curve. The total content of phenolic compounds in the extracts was expressed as gallic acid equivalents (GAE) mg/g of dry extract.

### Total flavonoid content

The aluminium chloride colorimetric method was used to estimate total flavonoid content in plant extract and fractions [29]. Quercetin was used to prepare the calibration curve. Briefly, 0.5 mL standard/extract was mixed with 1.5 mL methanol, 0.1 mL of 10% aluminum chloride, 0.1 mL of 1 M potassium acetate and 2.8 mL of distilled water. The reaction mixture was incubated at room temperature for 30 min. The absorbance was measured at 415 nm with a UV-spectrophotometer. Aluminium chloride was substituted by the same amount of distilled water in blank. The concentration of flavonoid was determined from the standard quercetin calibration curve. The total flavonoid content of the extract and fractions was expressed as quercetin equivalents (QE) mg/g of dry extract.

### Antioxidant activity

#### DPPH radical scavenging activity

The free radical scavenging activity of the extract and fractions was evaluated using the stable DPPH free radical. One mL of 0.1 mM DPPH solution in methanol was added to 1.0 mL of standard/extract solution at different concentrations. The mixture was incubated for 20 min and the absorbance recorded at 517 nm [30]. Ascorbic acid was used as positive control. DPPH radical scavenging activity was calculated using the formula: Percent scavenging = ((A_o_ – A_t_)/A_o_) × 100; where A_o_ = Absorbance of control (without extract) and A_t_ = Absorbance of sample. All determinations were carried out in triplicate.

#### ABTS radical scavenging activity

ABTS free radical was generated by reacting 7 mM ABTS solution with 2.45 mM potassium persulphate. The mixture was allowed to stand for 15 h in dark at room temperature. ABTS solution was diluted with methanol to obtain the absorbance of 0.7 ± 0.2 units at 750 nm. The standard/extract solutions were prepared at different concentrations in methanol and 20 μL of test solutions were added to 180 μL of ABTS free radical solution. The absorbance was measured after 20 minutes incubation at 750 nm. Ascorbic acid was used as positive control. The ABTS free radical scavenging activity was calculated using the formula: Percent scavenging = ((A_o_ – A_t_)/A_o_) × 100; where A_o_ = Absorbance of control (without extract) and A_t_ = Absorbance of sample [31]. All the tests were performed in triplicate.

#### Nitric oxide scavenging activity

Nitric oxide scavenging activity was estimated by using the Griess reagent assay. Briefly 2.0 mL of 10 mM sodium nitroprusside was mixed with 0.5 mL phosphate buffered saline (PBS) and 0.5 mL of standard/extract solutions at different concentrations. The mixture was incubated at 25°C for 150 min. The reaction mixture (0.5 mL) was incubated with 1 mL sulphanilic acid reagent (0.33% sulphanilic acid in 20% glacial acetic acid) for 5 min followed by addition of 1 mL 0.1% naphthyl ethylene diamine dihydrochloride. This incubation mixture was allowed to stand for 30 min and absorbance read at 540 nm. Curcumin was used as positive control. Percentage scavenging was calculated by the following formula: Percent scavenging = ((A_o_ – A_t_)/A_o_) × 100; where A_o_ = Absorbance of control (without extract) and A_t_ = Absorbance of sample [32]. All the tests were performed in triplicate.

#### Iron chelating activity

The 1, l0-Phenanthroline-iron (III) reagent was prepared by mixing 0.198 g of l, l0-phenanthroline monohydrate, 2 mL of 1 M hydrochloric acid and 0.16 g of ferric ammonium sulphate in 100 mL water. Briefly, 0.2 mL standard/extracts were mixed with 0.2 mL 1, l0-phenanthroline-iron (III) reagent, 0.6 mL methanol and 4 mL water. The solutions were incubated at 50°C for 30 min and absorbance read at 510 nm. Ascorbic acid was used as positive control. A higher absorbance indicated higher iron chelating activity. Percentage scavenging was calculated by using the following formula: Percent scavenging = ((A_t_ – A_o_)/A_t_) × 100; where A_o_ = Absorbance of control (without extract) and A_t_ = Absorbance of sample [33,34]. All the tests were performed in triplicate.

#### Total antioxidant capacity

The total antioxidant capacities of the extract and fractions of *N. jatamansi* were determined using phosphomolybdenum method. Briefly, 0.1 mL of standard/extract solution was mixed with 0.3 mL of reagent solution (0.6 M sulfuric acid, 28 mM sodium phosphate and 4 mM ammonium molybdate) and incubated at 95°C for 90 min. The mixture was cooled down to room temperature and absorbance recorded at 695 nm. The blank solution contained all the reagents except the test sample. Ascorbic acid was used to plot the standard curve. The results were expressed as ascorbic acid equivalents [35]. All the tests were performed in triplicate.

### Cell culture

Estrogen receptor (ER)-positive MCF-7 and ER-negative MDA-MB-231 breast cancer cells were procured from National Centre for Cell Science, Pune, India. The cells were cultured in Dulbecco’s Minimum Essential Medium (DMEM) with 10% fetal bovine serum (FBS) and 50 μg/mL gentamicin. The cells were incubated at 37°C in CO_2_ incubator in an atmosphere of humidified 5% CO_2_ and 95% air. The cells were maintained by sub-culturing in 25 cm^2^ tissue culture flasks. Cells growing in the exponential phase were used for cell viability assay.

### Cell viability by MTT assay

MTT assay was used to determine the inhibition of cancer cell proliferation by extract and fractions of *N. jatamansi.* Exponentially growing MCF-7 and MDA-MB-231 cells were seeded into 96-well plates (10^4^ cells/well in 100 μL of media) and allowed to attach for 24 h. Test extract/fractions were prepared in 0.1% DMSO and serially diluted with media to obtain appropriate concentrations. Cells were treated with different concentrations of extract/fractions and incubated for 48 h. Cells in the control group received only media containing 0.1% DMSO. The test compound containing media was removed and washed with 200 μL of PBS followed by addition of 20 μL of MTT reagent (5 mg/mL MTT in PBS) and incubated for 4 h at 37°C. The medium was removed and 100 μL DMSO was added and the absorbance measured using a micro plate reader at 540 nm followed by the calculation of percentage viability [36]. Percentage cell viability = 100 - [((A_o_-A_t_)/A_o_) × 100], where A_o_ = Absorbance of cells treated with 0.1% DMSO medium, A_t_ = Absorbance of cells treated with extract/fractions. 0.1% (v/v) DMSO in medium was used as negative control. Each treatment was performed in triplicate. Doxorubicin was used as standard. IC_50_ values were calculated using dose response inhibition curves in Graph pad prism 5.

### Sulforhodamine B assay

The SRB assay was performed according to the method developed by Vichai and Kirtikara [37] in MDA-MB-231 cells. The method of plating and incubation were identical to MTT assay. After treatment with the extract/fractions, cells were fixed with 50% trichloroacetic acid at 4°C (50 μL/well) for 1 h. The plate was washed with tap water for five times, dried and stained with SRB dye (0.057% in 1% acetic acid) for 30 min and subsequently washed with 1% acetic acid to remove the unbound dye. Plate was air-dried and bound protein stain was solubilised with 100 μL 10 mM Tris base. The absorbance was recorded at 540 nm and percentage cell viability calculated as described for MTT assay.

### Analysis of cell cycle distribution profile

The effect of extract/fractions on cell cycle distribution was assessed by flow cytometry after propidium iodide staining [38]. MDA-MB-231 cells (1 × 10^5^ cells/well) were treated with NJM and NJDE at 20 μg/mL and NJPE and NJEA at 35 μg/mL for 48 h. Cells in the control group received only media containing 0.1% DMSO and doxorubicin was used as positive control. Cells were harvested, washed with PBS, fixed with ice-cold 70% ethanol and kept at 4°C for 12 h. The cells were again washed with cold PBS and stained with propidium iodide (5 μg/mL) solution containing 0.1 mg/mL RNase and incubated in dark for 30 min. The cellular DNA content was analyzed by flow cytometry (Becton Dickinson Accuri, San Diego, CA, USA) and percentage of cells determined in G_0_/G_1_, S, and G_2_/M phases of cell cycle using the BD software after exclusion of cellular debris and aggregates.

### Hoechst 33258 staining

Apoptotic activity of extract/fractions was determined by Hoechst 33258 staining as described by Harada *et al.* [39]. MDA-MB-231 cells (1 × 10^5^) cells were treated with NJM and NJDE at 20 μg/mL and NJPE and NJEA at 35 μg/mL for 48 h. Cells in the control group received only media containing 0.1% DMSO and doxorubicin was used as positive control. Cells were washed with PBS and fixed with 70% ethanol for 5 min. After fixation, cells were incubated with Hoechst 33258 stain in PBS (5 μg/mL) for 30 min at 37°C in the dark. Cells were thoroughly washed with PBS and examined under a fluorescent microscope with an excitation of 350 nm and emission of 460 nm. Apoptotic cells were identified by nuclear condensation, formation of membrane blebs and apoptotic bodies. The mean number of apoptotic cells was determined by counting apoptotic cells in six different fields.

### Clonogenic assay

MDA-MB-231 cells (400 cells/well) were added in a 6-well plate and treated with NJM and NJDE at 20 μg/mL and NJPE and NJEA at 35 μg/mL for 48 h. Cells in the control group received only media containing 0.1% DMSO and doxorubicin was used as positive control. The media was removed after treatment and cells were incubated with fresh media for 12 days, fixed with 70% ethanol and stained with crystal violet (0.5% in ethanol). Cell colonies with more than 50 cells were counted [40]. The treatments were carried out in triplicate.

### Quantitative analysis of lupeol and β-sitosterol in extract/fractions by HPTLC

A validated high performance thin layer chromatography (HPTLC) method was used to detect and quantify the content of lupeol and β-sitosterol [41] in extract/fractions of *N. jatamansi*. The standard solutions were prepared at a concentration of 100 μg/mL and extract/fractions prepared at concentration of 2 mg/mL in methanol. The samples were spotted on pre-coated silica gel GF_254_ plates (20 cm × 10 cm with 0.2 mm thickness, E. Merck, Germany) using a Camag Linomat 5 applicator under nitrogen gas flow. Ten microlitres of sample solutions were applied in the form of bands of 8 mm width in duplicate. The mobile phase composed of toluene:methanol (9:1, v/v) and kept in Camag twin trough chamber for saturation for 20 min. The plate was developed up to 75 mm from the point of application in linear ascending manner. Scanning was performed with Scanner-3 with slit dimensions of 6 mm × 0.45 mm. After development, the plate was dried and derivatized with anisaldehyde-sulfuric acid reagent at 110°C for 10 min and scanned at 600 nm. The peak areas were recorded and percentage of lupeol and β-sitosterol were calculated.

### Statistical analyses

The results are expressed as mean ± standard error of mean (SEM) of three replicate determinations and then analyzed by Graph pad prism 5. One way analysis of variance (ANOVA) and post-hoc Tukeys test were used to determine the differences among the means. Pearson’s correlation analysis was used to determine the correlation between total phenolic and flavonoid content, and antioxidant activities. A value of *P* < 0.05 was considered to be statistically significant.

## Results and discussion

The present study aimed to investigate the antioxidant and anticancer activity of the whole extract and subsequent fractions of *N. jatamansi* in breast cancer cells. Few research groups have evaluated the cytotoxic activity of *N. jatamansi* extract in various cancer cell lines [23-25], however, the mechanism of action of antiproliferative activity has not been investigated in breast cancer till date. These prompted us to investigate the anticancer potential of NJM extract and identify the most bioactive fraction with antiproliferative potential against ER-positive and ER-negative breast carcinoma cells and decipher the possible mechanisms of antiproliferative activity. In addition, the antioxidant activity and phytochemical analyses was carried out for NJM/fractions to further its use as an antioxidant and chemopreventive agent.

### Phytochemical analysis

The extraction yield, total phenolic and flavonoid content in the extract and fractions of *N. jatamansi* are summarized in Table 1.

**Table 1:** **Percent yield, total phenolic and flavonoid content of extract and fractions of** ***N. jatamansi***

**Extract/fraction**	**Percent yield**	**Total phenolic content**	**Total flavonoid content**
		**(mg GAE/g of plant extract)**	**(mg QE/g of plant extract)**
NJM	8.40	17.97 ± 0.22^a^	1.11 ± 0.04^a^
NJPE	1.50	4.66 ± 0.05^b^	ND
NJDE	70.19	16.56 ± 0.09^c^	6.21 ± 0.06^b^
NJEA	10.75	43.31 ± 0.23^d^	2.51 ± 0.005^c^
NJAQ	15.66	13.52 ± 0.02^e^	0.02 ± 0.005^d^

All the values are expressed as mean ± SEM (n = 3). ND: Not detected.

^a-e^Column wise values with different superscripts of this type indicate significant difference (*P* < 0.05) .

Phytochemical analysis of total NJM extract revealed the presence of sterols, triterpenoids, phenols, flavonoids, alkaloids, saponins, tannins, fixed oils and fats. Liebermann Burchard and Salkowski’s test were positive for NJPE, NJDE, and NJEA exhibiting the presence of sterols and triterpenoids. NJDE, NJEA and NJAQ showed a strong presence of phenols in the ferric chloride test. NJAQ exhibited the presence of tannins in the lead acetate test. The Shinoda test was positive for NJDE and NJEA suggesting the presence of flavonols (isoflavones), flavones, flavonones, flavononols. Dragendorff and Mayer’s test were positive for NJEA and NJAQ suggesting the presence of alkaloids. The foam test exhibited the presence of saponins in NJAQ.

Phenols are an important class of antioxidants due to their ideal structural chemistry for free-radical scavenging activity. They can act as reducing agents, metal chelators and free radical quenchers by donating an electron or hydrogen atom to free radicals [42]. The highest amount of phenolics was detected in NJEA (43.31 ± 0.23 mg GAE/g of plant extract) which was found to be significantly higher (*P* < 0.05) than other plant fractions. The non-polar NJPE had the least phenolic content (4.66 ± 0.05 mg GAE/g of plant extract). Results were calculated from standard gallic acid calibration curve (R^2^ = 0.9980).

Flavonoids form the largest group of natural phenolic compounds and possess excellent free radical scavenging and antioxidant properties [43]. The total flavonoid content was determined using a linear calibration curve of quercetin (R^2^ = 0.999) using the aluminium chloride method. NJDE was found to contain the highest quantity of flavonoids (6.21 ± 0.06 mg QE/g of plant extract) followed by NJEA (2.51 ± 0.005 mg QE/g of plant extract). No flavonoids were detected in the non-polar NJPE fraction.

### Antioxidant activity

ROS/RNS have a well-documented role in numerous ailments including cancer and despite the presence of endogenous defence mechanisms, an imbalance in the redox system leads to oxidative stress and damage to cellular molecules leading to carcinogenesis [8,44]. Owing to the presence of phenols and flavonoids in NJM/fractions, we evaluated the free radical scavenging activity using suitable *in vitro* models which would further its use as an adjuvant in chemoprevention.

### *In vitro* free radical scavenging activity

NJM/fractions showed dose-dependent scavenging activity of DPPH and ABTS free radicals. The highest free radical scavenging was observed for NJEA with an IC_50_ of 99.17 ± 3.76 μg/mL in DPPH and 82.10 ± 1.70 μg/mL in ABTS assay (Table 2). Correlation analysis revealed a moderate correlation (R = −0.7786) between total phenolic content and DPPH scavenging activity. A strong and significant (R = −0.9680, *P* < 0.05) correlation was observed between total phenolic content and ABTS scavenging indicating that phenolic compounds are primarily responsible for ABTS radical scavenging activity (Table 3).

**Table 2:** **Free radical scavenging and antioxidant capacity of extract and fractions of** ***N. jatamansi***

**Extract/fraction**	**DPPH scavenging**	**ABTS scavenging**	**Nitric oxide scavenging**	**Iron chelation**	**Total antioxidant capacity** ^**g**^
	**IC**_**50**_ **(μg/mL)**				
NJM	314.05 ± 5.76^a^	102.51 ± 4.36^a^	477.37 ± 9.41^a^	40.06 ± 0.37^a^	270.00 ± 6.12^a^
NJPE	>1000	>1000	NA	78.32 ± 1.03^b^	163.70 ± 1.48^b^
NJDE	236.45 ± 6.33^b^	111.73 ± 4.67^a^	260.37 ± 5.00^b^	21.63 ± 0.78^c^	448.15 ± 1.34^c^
NJEA	99.17 ± 3.76^c^	82.10 ± 1.70^b^	144.23 ± 8.30^c^	8.51 ± 1.03^d^	680.74 ± 1.34^d^
NJAQ	334.95 ± 0.94^a^	108.40 ± 3.59^a^	194.43 ± 8.54^d^	190.15 ± 0.53^e^	37.04 ± 0.37^e^
Ascorbic acid	7.12 ± 0.31^d^	2.05 ± 0.28^c^	-	1.19 ± 0.01^f^	-
Curcumin	-	-	21.59 ± 0.98^e^	-	-

All the values are expressed as mean ± SEM (n = 3). NA: Not active.

^a-f^Column wise values with different superscripts of this type indicate significant difference (*P* < 0.05).

^g^Total antioxidant capacity expressed as μg ascorbic acid equivalents/mg extract.

**Table 3:** **Correlation (R) between total phenolic and total flavonoid content of extract and fractions of** ***N. jatamansi*** **versus IC**_**50**_ **values of various antioxidant assays**

	**Correlation coefficient (R)**				
	**DPPH scavenging**	**ABTS scavenging**	**Nitric oxide scavenging**	**Iron chelation**	**Total antioxidant capacity**
Total phenolic content	−0.7786	−0.9680^a^	−0.4655	−0.5530	0.8396
Total flavonoid content	−0.5618	0.1741	−0.1160	−0.6401	0.5691

^a^p < 0.05, indicates statistically significant correlation.

The correlation coefficients between total flavonoid content and DPPH and ABTS radical scavenging assays were much lower as compared to total phenolic content. The total flavonoid content is a sum of flavones, flavonols and flavonones. However, the aluminium chloride method estimates only the content of flavones and flavonols [29,44,45] therefore, the total flavonoid content could be underestimated resulting in lower correlation coefficients with free radical scavenging assays.

### Nitric oxide scavenging activity

Nitric oxide plays an important role in several inflammatory diseases and carcinogenesis. In the present study, NJM and fractions were tested for their inhibitory effect on nitric oxide production using Griess reagent [46]. NJEA exhibited the highest nitric oxide scavenging potential in a dose-dependent manner with an IC_50_ of 144.23 ± 8.30 μg/mL (Table 2). Upon correlation analysis, the extract and fractions did not show a strong correlation between nitric oxide scavenging and total phenolic (R = −0.4655, *P* > 0.05) and flavonoid content (R = −0.1160, *P* > 0.05) suggesting the presence of other non-phenolic compounds with nitric oxide scavenging potential (Table 3).

### Iron chelation activity

Iron ions are known to catalyze the conversion of less reactive species like H_2_O_2_ or lipid peroxides to more reactive ones such as hydroxyl, peroxyl/alkoxyl radicals. The release of iron by cellular damage can accelerate oxidative damage hence, compounds with iron chelating ability can act as powerful antioxidants [47]. NJEA showed the highest ferric reduction potential (IC_50_ = 8.51 ± 1.03 μg/mL) which was significantly higher (*P* < 0.05) than the other fractions (Table 2). A moderate correlation was observed with total phenolic (R = −0.5530, *P* > 0.05) and flavonoid content (R = −0.6401, *P* > 0.05) indicating that other non-phenolic constituents are also responsible for the ferric reduction potential (Table 3).

### Total antioxidant capacity

Total antioxidant activity of extract and fractions were analyzed by the formation of phosphomolybdenum complex. NJEA showed the highest antioxidant capacity equal to 680.74 ± 1.34 AAE/mg extract followed by NJDE, NJM, NJPE and NJAQ (Table 2). Correlation analysis revealed that the antioxidant capacity of the extract and fractions exhibited a strong correlation (R = 0.8396, *P* > 0.05) with total phenolic content suggesting that phenolic compounds are responsible for the antioxidant capacity of the extracts (Table 3).

### Anticancer activity

#### Antiproliferative activity of NJM/fractions in MCF-7 and MDA-MB-231 cells by MTT assay

The antiproliferative potential of NJM/fractions was determined by MTT assay on two breast cancer cell lines (MCF-7 and MDA-MB-231). The extract and fractions showed dose-dependent inhibition of cell proliferation in MCF-7 and MDA-MB-231 cell lines (data not shown). Interestingly, the highest toxicity was observed for NJM in both breast cancer cell lines indicating the synergistic behaviour of compounds present in the whole extract. The IC_50_ values for NJM were found to be 58.01 ± 6.13 and 23.83 ± 0.69 μg/mL in MCF-7 and MDA-MB-231 cells respectively. The cytotoxicity of NJM to MDA-MB-231 cells was significantly (p < 0.05) higher than MCF-7 cells. All the fractions exhibited cytotoxicity to both cell lines with NJDE (IC_50_ = 25.04 ± 0.90 μg/mL) and NJPE (IC_50_ = 60.59 ± 4.78 μg/mL) being most effective in MDA-MB-231 and MCF-7 cells respectively (Table 4). The phenol-rich NJEA fraction also exhibited cytotoxicity to both breast cancer cells with IC_50_ values of 65.44 ± 4.63 μg/mL and 40.72 ± 5.22 μg/mL in MCF-7 and MDA-MB-231 cells respectively in MTT assay (Table 4).

**Table 4:** **IC**_**50**_ **values of extract and fractions of** ***N. jatamansi*** **and doxorubicin in ER (+) MCF-7 and ER (−) MDA-MB-231 human breast carcinoma cells**

**Extract/fraction**	**IC**_**50**_ **(μg/mL)**	
	**MCF-7**	**MDA-MB-231**
NJM	58.01 ± 6.13^a,g^	23.83 ± 0.69^a,g^
NJPE	60.59 ± 4.78^a,d^	38.25 ± 2.17^a,e^
NJDE	69.94 ± 3.92^a,d,e,h^	25.04 ± 0.90^a,e,f,h^
NJEA	65.44 ± 4.63^a,d,e^	40.72 ± 5.22^b,e,f^
NJAQ	141.35 ± 13.35^b^	117.13 ± 6.29^c^
Doxorubicin	2.20 ± 0.30^c^	0.31 ± 0.09^d^

All the values are expressed as mean ± SEM (n = 3). ^a-f^Column wise values with different superscripts of this type indicate significant difference (*P* < 0.05).

^g-h^Row wise values with different superscripts of this type indicate significant difference (*P* < 0.05).

#### Antiproliferative activity of NJM/fractions in MDA-MB-231 cells by SRB assay

In order to confirm the findings of MTT assay, SRB assay was carried out in MDA-MB-231 cells since some phytochemicals are known to interfere with MTT giving false positive results [48]. Comparable results were obtained in SRB assay with NJM exhibiting the highest cytotoxicity (IC_50_ = 23.34 ± 1.97 μg/mL) in MDA-MB-231 cells. The order of potency in SRB assay was found to be similar as MTT assay with NJDE being the most effective fraction in MDA-MB-231 cells with an IC_50_ of 23.80 ± 0.66 μg/mL (Table 5).

**Table 5:** **IC**_**50**_ **values of extract and fractions of** ***N. jatamansi*** **and doxorubicin in ER (−) MDA-MB-231 human breast carcinoma cells by SRB assay**

**Extract/fraction**	**IC**_**50**_ **(μg/mL)**
NJM	23.34 ± 1.97^a^
NJPE	40.83 ± 2.36^a,d^
NJDE	23.80 ± 0.66^a,d,e^
NJEA	38.67 ± 0.61^a,d,e^
NJAQ	123.10 ± 9.33^b^
Doxorubicin	0.38 ± 0.07^c^

All the values are expressed as mean ± SEM (n = 3). ^a-e^Column wise values with different superscripts of this type indicate significant difference (*P* < 0.05).

Since the extract/fractions showed more promising activity in MDA-MB-231 cells, further mechanistic studies were carried out to determine whether growth inhibition was due to cell cycle arrest and/or induction of apoptosis in MDA-MB-231 cells. Based on the results obtained in cytotoxicity assays, NJM, NJPE, NJDE, and NJEA were investigated for their mode of action in MDA-MB-231 cells.

#### NJM/fractions induce cell cycle arrest in MDA-MB-231 cells by flow cytometry

**Figure 1:**
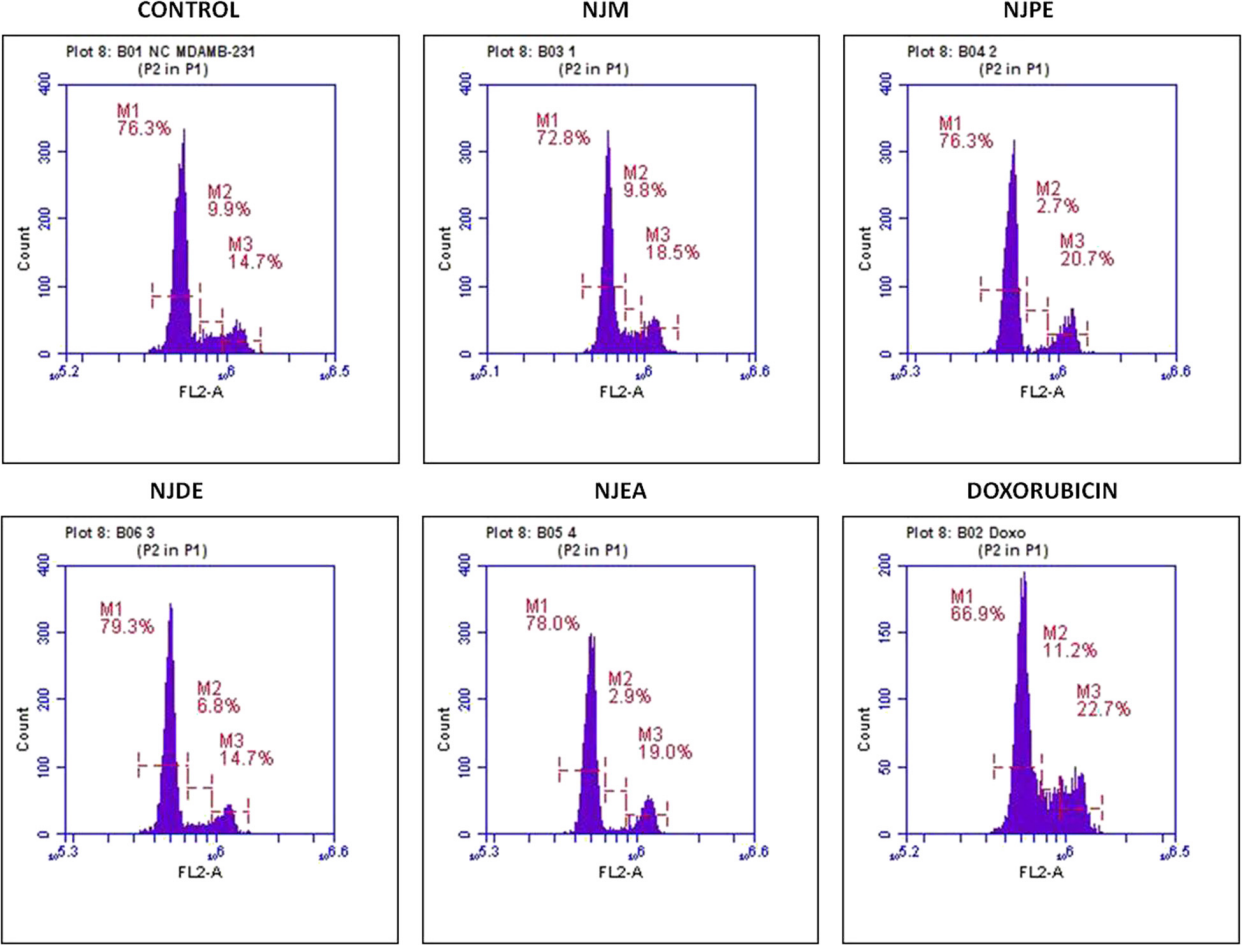
**NJM/fractions induce cell cycle arrest in MDA-MB-231 cells analyzed by flow cytometry.** MDA-MB-231 cells were treated with 0.1% DMSO (negative control), NJM, NJPE, NJDE, NJEA, and doxorubicin (positive control) for 48 h. The cells were fixed, stained with propidium iodide and analyzed by flow cytometry. The histograms represent relative cell DNA content measured in three independent experiments.

The antiproliferative effects of NJM and active fractions were investigated on the cell cycle distribution in MDA-MB-231 cells by propidium iodide staining and flow cytometry. MDA-MB-231 cells treated with NJM showed an increase in the percentage of cells in G_2_/M phase as compared to control cells treated with 0.1% DMSO (18.5% vs 14.7%). Interestingly, treatment with NJDE increased the proportion of cells in G_0_/G_1_ phase when compared to negative control cells (79.3% vs 76.3%) while NJPE and NJEA showed G_2_/M arrest as compared to negative control cells (20.7% and 19% respectively vs 14.7%) (Figure 1). Doxorubicin showed a prominent increase in cells in G_2_/M phase (22.7% vs 14.7%) which was in accordance with the published data that doxorubicin causes cell cycle arrest at G_2_/M phase in MDA-MB-231 cells [49].

#### NJM/fractions induce apoptosis in MDA-MB-231 cells by Hoechst 33258 staining

**Figure 2:**
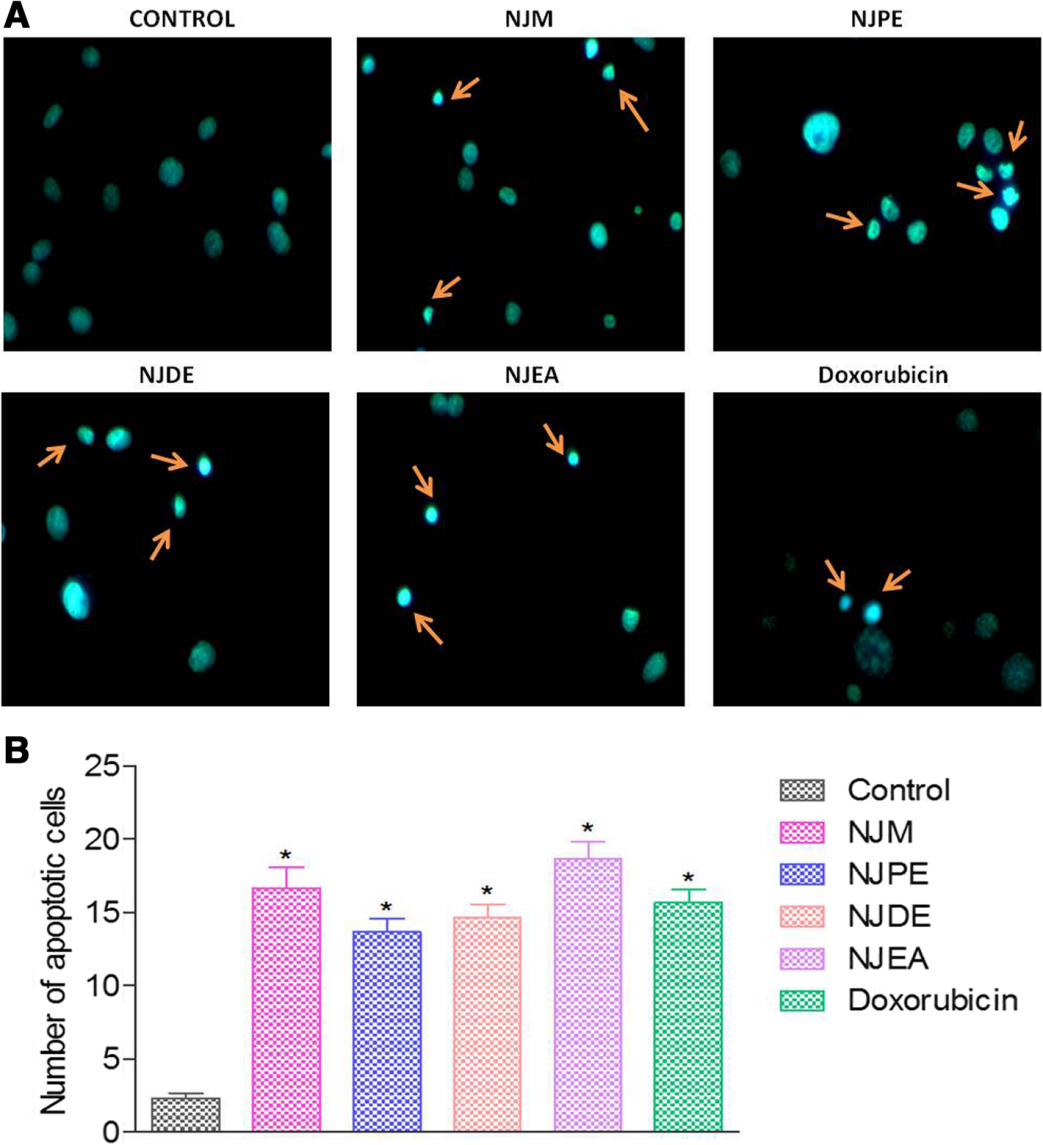
**NJM/fractions induce apoptosis in MDA-MB-231 cells by Hoechst 33258 staining.** MDA-MB-231 cells were treated with 0.1% DMSO (negative control), NJM, NJPE, NJDE, NJEA, and doxorubicin (positive control) for 48 h. The cells were fixed, stained with Hoechst 33258 and observed under fluorescent microscope at 40X. **(A)** Representative images of cells after treatment for 48 h. Apoptotic morphology was confirmed by nuclear condensation, formation of membrane blebs, round apoptotic bodies (orange arrows) **(B)** Number of apoptotic cells were estimated by counting apoptotic cells in six different fields. Results are expressed as mean ± SEM. *P < 0.001 compared with negative control. The treatments were carried out in triplicate.

To confirm the apoptotic effects of NJM/fractions, Hoechst 33258 stain was used to study the morphological changes in MDA-MB-231 cells under microscope. After treatment with NJM/fractions for 48 h, significant (*P* < 0.001) increase in apoptotic cells were observed as compared to negative control cells which showed regular contours with uniform fluorescence intensity. The treated cells exhibited characteristic apoptotic changes for example cell shrinkage, nuclear condensation, and formation of round apoptotic bodies which appeared as round spherical beads (Figure 2A,B). Our findings suggest that NJM, NJPE, NJDE and NJEA cause cell death by inducing apoptosis in MDA-MB-231 cells which is a highly desirable feature of anticancer agents. Further studies are warranted to evaluate whether the extrinsic or intrinsic pathways are induced by NJM/fractions.

#### NJM/fractions reduce clonogenic capacity of MDA-MB-231 cells

**Figure 3:**
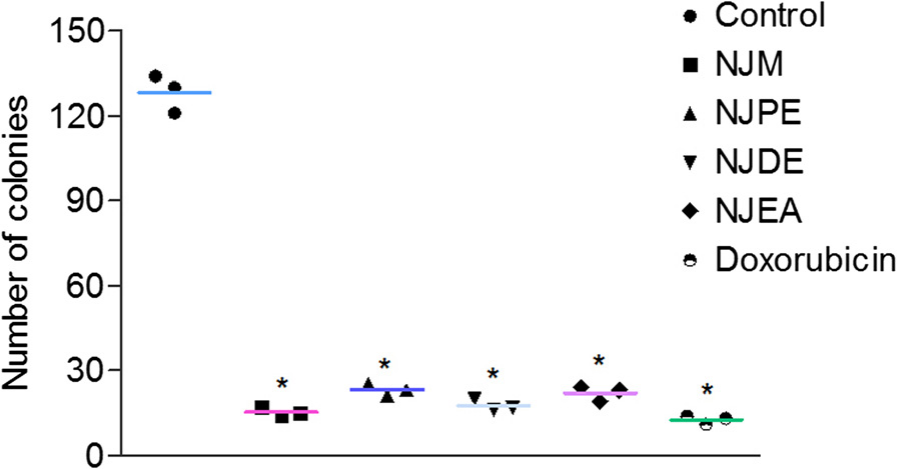
**NJM/fractions reduce clonogenic capacity of MDA-MB-231 cells.** MDA-MB-231 cells were treated with 0.1% DMSO (negative control), NJM, NJPE, NJDE, NJEA, and doxorubicin (positive control) for 48 h. After 12 days, cells were stained with crystal violet (0.5% in ethanol) and cell colonies (>50 cells/colony) counted. Results are expressed as mean ± SEM. *P < 0.001 compared with negative control. The treatments were carried out in triplicate.

Clonogenic assay is considered as the gold standard to determine the anticancer activity of drugs. NJM and fractions, NJPE, NJDE, and NJEA significantly (*P* < 0.001) reduced the colony formation of MDA-MB-231 cells over a period of 12 days thereby suggesting the long-term antiproliferative effect of NJM/fractions (Figure 3). Owing to their ability to inhibit colony formation of MDA-MB-231 cells, we speculate that NJM/fractions could significantly contribute to the reduction of metastases.

#### Quantification of lupeol and β-sitosterol in NJM/fractions by HPTLC

**Figure 4:**
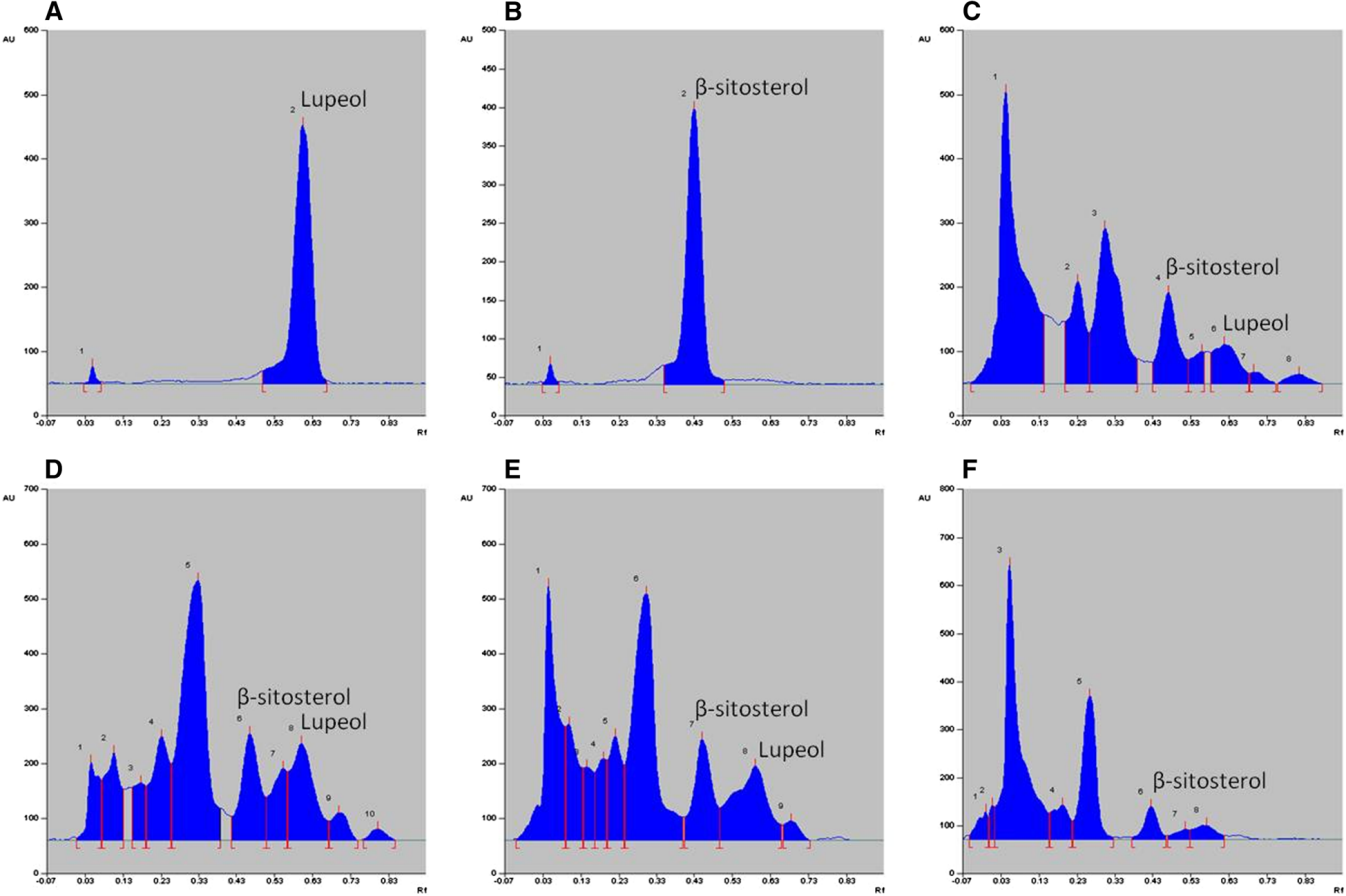
**HPTLC analysis of NJM/fractions with lupeol and β-sitosterol as standards.** HPTLC chromatographic profile of **(A)** Standard lupeol (R_f_ value = 0.67) **(B)** Standard β-sitosterol (R_f_ value = 0.48) **(C)** NJM **(D)** NJPE **(E)** NJDE **(F)** NJEA (R_f_ value of 0.67 ± 0.02 indicates lupeol and 0.48 ± 0.02 indicates β-sitosterol).

HPTLC analysis of the extract/fractions of *N. jatamansi* confirmed the presence of lupeol and β-sitosterol. Total NJM was found to contain 1.04% and 2.13% w/w lupeol and β-sitosterol respectively. Among the fractions, NJPE and NJDE were found to be enriched with 3.10% and 3.04% lupeol and 3.09% and 2.81% β-sitosterol respectively. Lupeol was not detected in NJEA and NJAQ and they showed relatively lower content of β-sitosterol (0.78% and 0.36%) (Figure 4). Our results were in accordance with previous studies that have reported the presence of lupeol and β-sitosterol in non-polar extract and oil of roots of *N. jatamansi* [16,50].

The anticancer activity of the identified compounds, lupeol and β-sitosterol has been reported previously. Lupeol is a pentacyclic triterpene that exerts antitumor activity by cell cycle regulation and inducing apoptosis. It is reported to inhibit the proliferation of MCF-7, MDA-MB-231, and other breast cancer cells in a dose and time-dependent manner [51,52]. It also causes G_2_/M arrest in prostate cancer cells which is mediated through *cyclin-B*-regulated signalling pathway [53]. Further, lupeol induced G_2_/M phase arrest and apoptosis in DMBA-induced carcinogenesis with upregulation of *Bax* and *caspase-3* and downregulation of *bcl-2* and *survivin* genes [54]. The NJM extract, NJPE and NJDE fractions were found to be enriched with lupeol which might have contributed to the anticancer activity of the extract/fractions. In addition, recent studies have confirmed the cytotoxic effect of β-sitosterol in MDA-MB-231 cells at concentration of 16 μmol/L which is mediated through induction of apoptosis by upregulation of bax/bcl-2 ratio, downregualtion of IAP family and caspase activation [55]. Moreover, another group confirmed the cytotoxic and pro-apoptotic effect of β-sitosterol in MDA-MB-231 cells providing valuable insight into the chemopreventive and therapeutic efficacy of β-sitosterol [56]. Overall, lupeol and β-sitosterol are reported to have multi-target action with immense anticancer potential modulating key signalling pathways that are implicated in various types of cancer, modulation of antioxidant enzyme levels in disease states, and reducing free radical generation [52,57]. Hence, these compounds can serve as excellent chemopreventive as well as therapeutic agents. As mentioned elsewhere in the article, NJDE exhibited the highest anticancer activity in MDA-MB-231 cells which could be attributed to the presence of lupeol and β-sitosterol along with the presence of other sesquiterpenes since the rhizomes are considered to be rich in jatamanshic acid, jatamansone, patchouli alcohol, nor-seychelanone, seychellen, alpha and beta patchoulene, valeranone, valeranal, nardol, calarenol, and nardostachone [16]. Sesquiterpenes are a promising class of natural compounds in cancer drug discovery with 3 compounds; artemisinin, thapsigargin and parthenolide currently being evaluated in clinical trials [58]. Other anticancer terpenoids from *N. jatamansi* include ursolic acid and 3-O-arabinosyl ursolic acid isolated from the cholorform:methanol fraction that showed cytotoxicity in lung (A-549), prostate (DU-145), breast cancer (MCF-7), and neuroblastoma (SK-N-SH) cells (IC_50_: 18–32 mM) [25]. Hence, the anticancer activity of the whole extract NJM and NJDE fraction against breast cancer cells could be attributed to the presence of a variety of compounds. Currently, further detailed characterization is being carried out in our laboratory to identify the active compounds and further study their mechanisms of action in breast cancer.

## Conclusion

Our results suggest that *N. jatamansi* may serve as an excellent lead for the development of anticancer agents for breast cancer particularly ER-negative breast carcinoma. It is an accessible source of natural antioxidants with considerable health benefits. Further work to elucidate the mechanism of action of cell death in breast cancer and to identify the active constituents is under study in our laboratory.

## Abbreviations

DPPH: 1,1-diphenyl-2-picrylhydrazyl
ABTS: 2,2′-azino-bis(3-ethylbenzothiazoline-6-sulfonic acid) diammonium salt
MTT: 3-(4,5-dimethylthiazolyl-2-yl)-2,5-diphenyl tetrazolium bromide
SRB: Sulforhodamine B
GAE: Gallic acid equivalents
QE: Quercetin equivalents
NJM: Methanol extract of *N. jatamansi*
ROS: Reactive oxygen species
RNS: Reactive nitrogen species
NJPE: Petroleum ether extract of *N. jatamansi*
NJDE: Diethyl ether fraction of *N. jatamansi*
NJEA: Ethyl acetate fraction of *N. jatamansi*
NJAQ: Aqueous fraction of *N. jatamansi*
ER: Estrogen receptor
DMEM: Dulbecco’s minimum essential medium
FBS: Fetal bovine serum
DMSO: Dimethyl sulfoxide
ANOVA: One way analysis of variance
